# Pneumopyopericardium mimicking an inferior ST elevation myocardial infarction with regional electrocardiogram changes: a case report

**DOI:** 10.1186/s13104-015-1143-7

**Published:** 2015-04-30

**Authors:** Eranda Chamara Ratnayake, Sandamali Premaratne, Niroshan Lokunarangoda, Sanduni Fernando, Nilanthi Fernando, Chandrike Ponnamperuma, W Samuel Santharaj

**Affiliations:** Institute of Cardiology, National Hospital of Sri Lanka, Regent Street, Colombo 7, Sri Lanka

**Keywords:** Pneumopyopericardium, Pneumopericardium, Mediastinitis, Myocardial infarction, STEMI

## Abstract

**Background:**

Pneumopyopericardium is a rare disease with poor prognosis. The usual presentation is with fever, shortness of breath and haemodynamic compromise. The Electrocardiogram changes associated with this disease entity would be similar to pericarditis such as concave shaped ST elevations in all leads with PR sagging. Pneumopyopericardium mimicking an acute ST Elevation Myocardial Infarction, with regional Electrocardiogram changes has hitherto not been described in world literature.

**Case Presentation:**

We describe the case of a 48 year old native Sri Lankan man, presenting with chest pain and Electrocardiogram changes compatible with an Acute ST Elevation Myocardial Infarction, subsequently found to have Pneumopyopericardium secondary to an oesophageal tear. Retrospective history revealed repetitive vomiting due to heavy alcohol consumption, prior to presentation. It unfortunately led to a fatal outcome.

**Conclusion:**

Pneumopyopericardium may mimic an acute ST elevation myocardial infarction with associated regional Electrocardiogram changes. A high degree of suspicion should be maintained and an adequate history should always be obtained prior to any intervention in all ST Elevation Myocardial Infarction patients.

## Background

Cardiovascular disease is the leading cause of mortality worldwide [1]. Pericardial disease and effusions account for approximately 6-7% of cardiac disease in the adult population [2] of which Pneumopyopericardium is a seldom reported disease entity. It was first described by Hallin in 1863 [3] and since then less than 20 cases have been reported in world literature. The presentation is often preceded by a traumatic or non-traumatic ulceration of the oesophagus or stomach. Presenting symptoms of pneumopyopericardium include but are not limited to fever, pleuritic chest pain, shortness of breath associated with significant haemodynamic compromise [4]. The commonest precipitating cause is a non-traumatic oesophageal ulcer or carcinoma [4]. Electrocardiogram (ECG) changes associated with the disease are comparable to generalized pericarditis, with concave shaped ST elevations in all leads with PR segment sagging [5]. A presentation mimicking an Acute ST Elevation Myocardial Infarction (STEMI) with regional electrocardiogram changes has hitherto not been described in published world literature.

## Case presentation

**Figure 1:**
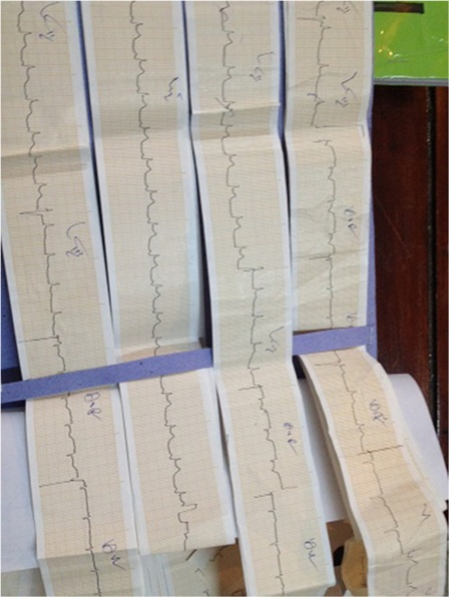
Electrocardiogram – Initial Electrocardiogram of the patient showing ST elevations in the Inferior Leads with Reciprocal ST depressions in lateral leads.

**Figure 2:**
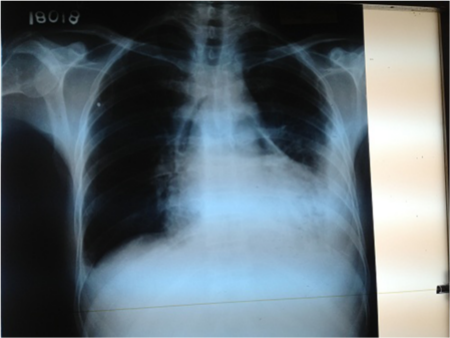
Chest X-Ray – Chest X-Ray – Anterior-Posterior View of the patient showing air and fluid around the heart.

**Figure 3:**
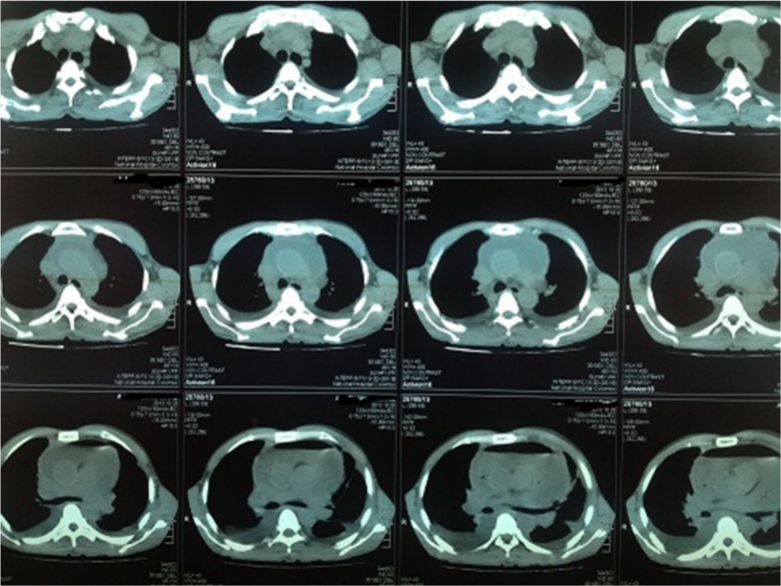
Contrast Computer Tomography Chest – Contrast Computed Tomography Chest showing air and fluid around the heart.

**Figure 4:**
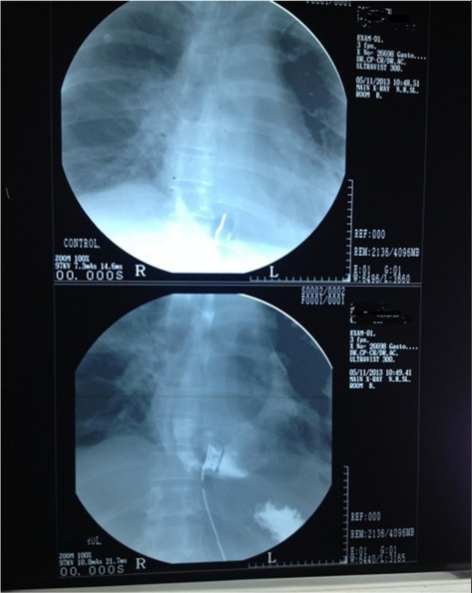
Gastrograffin Test – Gastrograffin test showing lower oesophageal rupture and covered stent placement.

A 48 year old Sinhalese man from Chillaw, Sri Lanka presented to his local hospital with a history of sudden onset retrosternal chest pain, shortness of breath and sweating. He was a heavy alcohol abuser, consuming 10 Units of Local Arrack (Ethanol) on a daily basis with occasional use of illicit alcohol (Methanol). An ECG taken at the local hospital is seen in Figure 1. It was suggestive of an Acute Inferior ST Elevation Myocardial Infarction. In the absence of any known complications and as interventional facilities were not available at that centre, he was thrombolysed with IV streptokinase. As the patient had persistent chest pain and unresolved ST segment changes, he was transferred to the National Hospital of Sri Lanka for further management. A retrospective history revealed the patient had repetitively vomited prior to his initial presentation preceded by heavy alcohol consumption. Examination revealed muffled heart sounds and a pericardial friction rub. An immediate bedside 2 Dimensional Echocardiogram was performed which had poor echo windows but a moderate pericardial effusion was identified with no clear evidence of tamponade. There was no significant regional wall motion abnormalities. A Chest X-Ray – Antero-Posterior View was performed (Figure 2) which was followed by a Contrast Computer Tomography Chest (Figure 3). An urgent pericardial aspiration evacuated 800 millilitres of frank pus and a diagnosis of pneumopyopericardium was made. In search of a possible aetiology, a Gastrograffin study was performed (Figure 4). An immediate surgical referral was made which was followed by endoscopic covered stent placement. Intravenous meropenem was commenced pending culture reports. Troponin I titres were negative. A coronary angiogram was not performed on the patient due to high risk and haemodynamic instability. Unfortunately, two days after treatment at the coronary care unit, the patient developed a cardiac arrest and expired.

## Discussion

Pneumopyopericardium is an uncommon disease entity, with less than 20 cases reported to date in world literature. Our case provides insight in to a rare and never before described presentation mimicking an acute ST elevation myocardial infarction with regional electrocardiogram changes. Although falsely managed as an acute coronary syndrome initially, a diagnosis of pneumopyopericardium was retrospectively made. A focused history prior to intervention may have predicted the disease, as the patient complained of persistent vomiting secondary to heavy alcohol abuse. Consistent with other reported cases, this patient was also found to have an oesophageal rupture. A possible cause for acute ST segment elevations in this scenario could be a localized myopericarditis, which has been reported previously [5]. Regional ST segment changes are known to occur in this circumstance, contrary to the generalized ECG changes associated with a more extensive pericarditis. A coronary angiogram was not performed on this patient to assess his coronary arteries as he was unstable haemodynamically and carried high mortality risk. However, an acute coronary syndrome secondary to significant coronary artery disease could be excluded by the negative Troponin titre and lack of significant regional wall motion abnormalities on 2 Dimensional echocardiography. Although timely intervention was initiated with a covered stent placed at the ruptured part of the oesophagus by the surgical team, overwhelming sepsis took over and the patient eventually had a fatal outcome. Poor prognosis following pneumopyopericadium is well documented in world literature [6].

## Conclusions

Pneumopyopericardium may present as an acute ST elevation myocardial infarction. A high degree of suspicion should be maintained in the correct clinical setting. The prognosis remains poor, despite maximal medical and surgical management for the condition.

## Consent

Written informed consent was obtained from the patient for publication of this Case Report and any accompanying images. A copy of the written consent is available for review by the Editor-in-Chief of this journal.

## Abbreviations

ECG: Electrocardiogram
STEMI: ST Elevation Myocardial Infarction
CXR: Chest X-Ray
CT: Computed tomography
